# Effects of endogenous neurotoxin quinolinic acid on reactive oxygen species production by Fenton reaction catalyzed by iron or copper

**DOI:** 10.1016/j.jorganchem.2015.01.030

**Published:** 2015-04-15

**Authors:** Lenka Kubicova, Franz Hadacek, Wolfram Weckwerth, Vladimir Chobot

**Affiliations:** a Division of Molecular Systems Biology, Department of Ecogenomics and Systems Biology, Faculty of Life Sciences, University of Vienna, Althanstrasse 14, Vienna A-1090, Austria; b Plant Biochemistry, Albrecht-von-Haller Institut, Georg-August-Universität Göttingen, Justus-von-Liebig-Weg 11, Göttingen D-37077, Germany

**Keywords:** Coordination complexes, Reactive oxygen species, Kynurenines, Mass spectrometry, Degenerative diseases, Neurotoxin

## Abstract

The tryptophan metabolite, quinolinic (2,3-pyridinedicarboxylic) acid, is known as an endogenous neurotoxin. Quinolinic acid can form coordination complexes with iron or copper. The effects of quinolinic acid on reactive oxygen species production in the presence of iron or copper were explored by a combination of chemical assays, classical site-specific and ascorbic acid-free variants of the deoxyribose degradation assay, and mass spectrometry (ESI–MS). Quinolinic acid showed evident antioxidant activity in chemical assays, but the effect was more pronounced in the presence of copper as transition metal catalyst than in presence of iron. Nano-ESI–MS confirmed the ability of quinolinic acid to form coordination complexes with iron(II) or copper(II) and quinolinic acid stability against oxidative attack by hydroxyl radicals. The results illustrate a highly milieu-dependent quinolinic acid chemistry when it enters reactions as competitive ligand.

## Highlights

• Quinolinic acid is considered as a neurotoxin but it can also act as an antioxidant.
• MS proves quinolinic acid's ability to form coordination complexes with Fe or Cu.
• Quinolinic acid showed a relative robustness when under oxidative attack.
• Quinolinic acid can protect cells suffering from the elevated oxidative stress.

## Graphical abstract

Quinolinic acid is a tryptophan metabolite known by its neurotoxicity. The ability of quinolinic acid to form coordination complexes with iron or copper and its robustness against hydroxyl radical attack was investigated by ESI–MS.

**Figure:**
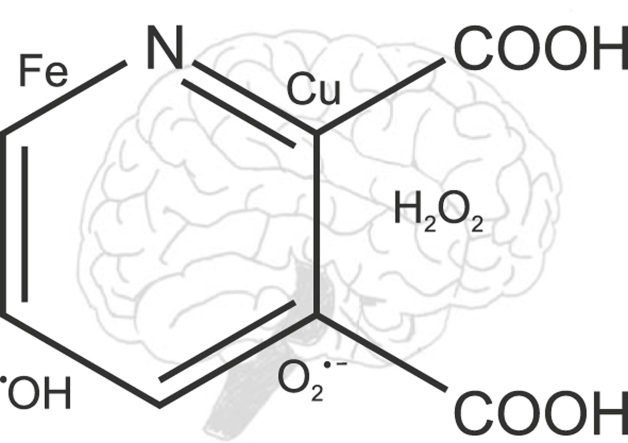

## Introduction

**Fig. 1:**
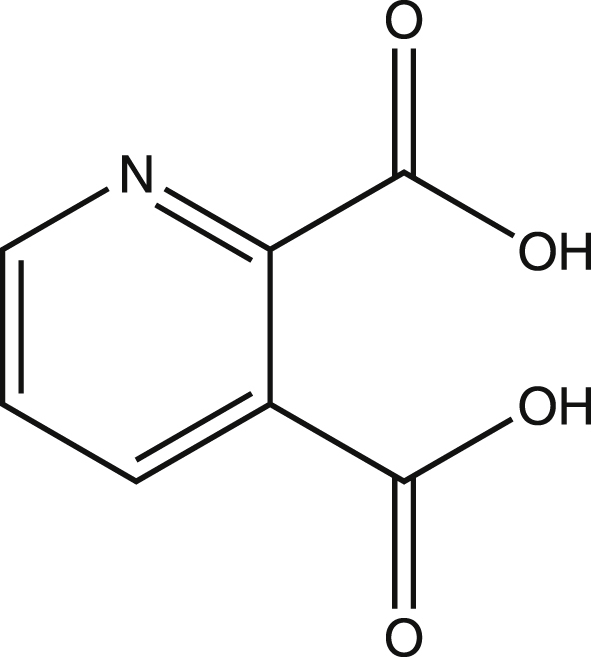
Structure of quinolinic acid (QUIN).

Quinolinic acid (2,3-pyridinedicarboxylic acid, QUIN) (Fig. 1) is a metabolic intermediate of tryptophan catabolism within the kynurenine pathway. A few decades ago, kynurenines were suggested to act as important endogenous modulators of various brain functions [1]. The ratio among QUIN, 3-hydroxykynurenine and kynurenic acid can affect cognitive performance and neuronal vulnerability, among others. Kynurenines can participate in many neurodegenerative diseases and neurological impairments associated with some infectious diseases, such as AIDS or brain malaria [2], [3]. Especially, QUIN has been explored extensively for its effects on brain functions [4], [5], [6]. A higher than physiological concentration (below 100 nM) has been found in brains or cerebrospinal fluids of patients suffering from Alzheimer's or Huntington's disease, amyotrophic lateral sclerosis, depression, autism and schizophrenia [5], [6]. Under pathological conditions, the QUIN level can rise up to 10–40 μM [7]. Therefore, QUIN is assumed to act as an endogenous neurotoxin. Generally, QUIN neurotoxicity is suspected to be caused partly by the over-excitation of the NMDA (*N*-methyl-d-aspartate) receptor and partly by elevated levels of cytotoxic reactive oxygen species (ROS) in the brain tissue [1], [4]. QUIN can affect ROS production by forming coordination complexes with iron [8], [9].

Increased concentrations of iron and/or copper were detected in the brains of patients suffering from Alzheimer's [10], [11], [12], Huntington's, Parkinson's [11], [12], and Wilson's [12] diseases. For example, the concentrations of iron and copper in the brains of patients with Alzheimer's disease were found about 0.9 mM for iron and 0.4 mM for copper. The concentrations were significantly higher compared to controls (iron 0.3 mM and copper 0.07 mM respectively) [10]. Both transition metals can enhance neurodegenerative processes because of their ability to initiate the Fenton reaction that results in forming the highly cytotoxic hydroxyl radical [13].

Besides various non-heme and heme coordination complexes with proteins, iron and copper can be liganded also by low-molecular weight metabolites. Such complexes can catalyze ROS production. In a previous study, we showed that QUIN can cause either pro- or antioxidant effects, depending on reaction conditions [14]. We proposed that QUIN can affect ROS formation in processes that aim at maintaining the redox homeodynamic equilibrium that is required for cellular signaling and metabolic functions [15]. Here we explored if QUIN can act as a competitive ligand of copper and iron, which occurs in elevated concentrations e.g. in cerebral Alzheimer's or senile plaques. This provides some insights about QUIN capability to affect oxidative degradation rates of biomolecules (detection molecule 2-deoxy-d-ribose). Redox cycling of iron and copper were compared in the classical site-specific and ascorbic acid-free variants of the deoxyribose degradation assay. Furthermore, QUIN coordination complex formation ability and stability against ROS oxidative degradation were explored by nanoelectrospray ionization LTQ Orbitrap mass spectrometry (nano-ESI–MS). These experiments aimed to provide mechanistic ideas about the possible QUIN chemistry in brain tissues.

## Material and methods

### Chemicals

All chemicals and solvents used were purchased from Sigma–Aldrich (Schnelldorf, Germany). Water had Milli-Q quality.

### QUIN effect on copper or iron catalyzed ascorbic acid-free variant of deoxyribose degradation assay

The phosphate buffer and water, which were used as solvents for the tested substances, FeSO_4_ or CuSO_4_, were degassed by argon for 10 min at least. QUIN was dissolved in aqueous KH_2_PO_4_/KOH buffer solution (30 mM, pH 7.4) and diluted serially; to 125 μL of this solution, 25 μL of a 52 mM 2-deoxy-d-ribose solution in the same buffer system, 50 μL of the buffer, and 25 μL of degassed aqueous CuSO_4_ or FeSO_4_ solution (100 μM) and 25 μL of 10.0 mM aqueous H_2_O_2_ solution were added. The final concentrations of QUIN were 2–500 μM. Blanks contained the full reaction mixtures except for 2-deoxy-d-ribose. Standard 1.5 mL sample vials (La-Pha-Pack, Werner Reifferscheidt GmbH, Langerwehe, Germany) were used as reaction vials. The mixtures were incubated at 27 °C for 1 h. Thereafter, 250 μL of 1.0% thiobarbituric acid dissolved in 3% trichloroacetic acid (w/v) was added to each vial to detect TBARS. The vials were heated in a water bath at 80 °C for 30 min. The reaction was stopped by transferring the vials into an ice water bath for 3 min. To extract the TBARS, 600 μL of 1-butanol was added, and the mixture was rigorously vortexed. The butanol layers of the vials, each 350 μL, were pipetted into flat bottomed 96 well plates (Greiner, Kremsmünster, Austria), and the absorbance was determined with a microplate reader (Tecan Infinite M200, Männedorf, Switzerland) at 532 nm. Experiments were performed in triplicate. Reaction mixtures lacking the test compound served as the positive control (100% TBARS).

### Copper-catalyzed site-specific deoxyribose degradation assay

The assay followed published procedure with minor modifications [16]. QUIN was dissolved in aqueous KH_2_PO_4_/KOH buffer solution (30 mM, pH 7.4) and diluted serially; to 125 μL of this solution, 25 μL of a 10.4 mM 2-deoxy-d-ribose solution in the same buffer system, 25 μL of the buffer, and 25 μL of degassed aqueous CuSO_4_ solution (100 μM) were added. Further, 25 μL of 10.0 mM aqueous H_2_O_2_ solution and 25 μL of 1.0 mM ascorbic acid in the buffer were added to start the Fenton reaction in the H_2_O_2_/Cu^II^/ascorbic acid reaction mixture. The final concentrations of QUIN were 2–500 μM. The samples were incubated at 27 °C for 1 h. Thiobarbituric acid reactive species (TBARS) were determined photometrically at 532 nm after reaction with thiobarbituric acid and subsequent extraction of the pink pigment with 1-butanol. The H_2_O_2_/Cu^II^/ascorbic acid reaction mixture without QUIN served as the positive control and represented 100% TBARS. Blanks contained the full reaction mixtures except for 2-deoxy-d-ribose and were determined in each experiment. Experiments were performed in triplicate.

### Sample preparation for the analysis of QUIN oxidation stability

The samples for ESI–MS analyses of QUIN oxidation stability were prepared as followed: QUIN was dissolved in aqueous ammonium bicarbonate buffer solution (10 mM, pH 7.4 adjusted with HCl) to 1 mM solution. To 500 μL of this solution, 300 μL of 10 mM aqueous ammonium bicarbonate buffer, 100 μL of degassed aqueous FeCl_2_ or CuCl_2_ solution (100 μM) and 100 μL of aqueous H_2_O_2_ solution (55 mM) were added. Standard 1.5 mL sample vials (La-Pha-Pack, Werner Reifferscheidt GmbH, Langerwehe, Germany) were used as reaction vials. The mixture was incubated at 27 °C. The reaction mixtures were analyzed in reaction times 0 h and 3 h. For the ESI–MS measurement, 50 μL of the reaction mixture was diluted with 450 μL of 0.2% (v/v) formic acid in methanol and borosilicate glass emitters gold sputter coated (50 mm length, 10 and 5 μm tip ID, DNU-MS GbR) filled with 5 μL of this mixture were used.

### Sample preparation for nano-ESI–MS coordination complex analysis

QUIN coordination complexes were measured according the method of Sarowar et al. [17] with minor modifications. The samples for the nano-ESI–MS analysis of QUIN complexes were prepared from a 1 mM stock solution of QUIN in degassed methanol by adding of appropriate amount of degassed aqueous FeCl_2_ or CuCl_2_ solution (500 μM) in QUIN:metal(II) molar ratios 1:2, 1:1, 2:1, 3:1, and 4:1. Before the measurement, the samples were diluted 1:10 or 1:100 with water/methanol (50:50, v:v) mixture. For the ESI–MS measurement, borosilicate glass emitters gold sputter coated (50 mm length, 10 and 5 μm tip ID, DNU-MS GbR) filled with 5 μL of this mixture were used.

### Nano-ESI–MS

MS analyses were performed out on a Thermo Electron LTQ-Orbitrap XL mass spectrometer equipped with a nanoelectrospray ion source (ThermoFisher Scientific, Bremen, Germany) and operated under Xcalibur software, in the positive ionization mode. The instrument was calibrated using the manufacturer's calibration standards. The Fourier transformed full scan mass spectra were acquired at a target value of 10^6^ ions with a resolution of 100,000 in the *m/z* range of 80–2000. In order to achieve even higher mass accuracy, a lock mass option was enabled and the cyclomethicone N5 ions generated in the electrospray process from ambient air (*m/z* = 371.101230) were used for internal recalibration in real time. This allowed mass accuracies of <1 ppm. Specific tune settings for the MS were as follows: spray voltage was set to 1.8 kV; capillary voltage was 45 V, tube lens offset 150 V and capillary temperature was set at 180 °C, no sheath gas and auxiliary gas were used. Theoretical masses and isotope relative intensities were calculated with ChemDoodle 7.0.2, iChemLabs, LLC, Somset, NY.

### Statistical analysis

Statgraphics Centurion XVI (Statistical Graphics Corp., Rockville, MD, USA) was used to perform analyses of variance (ANOVA) employing 95% Duncan's multiple range *post hoc* test.

## Results and discussion

**Fig. 2:**
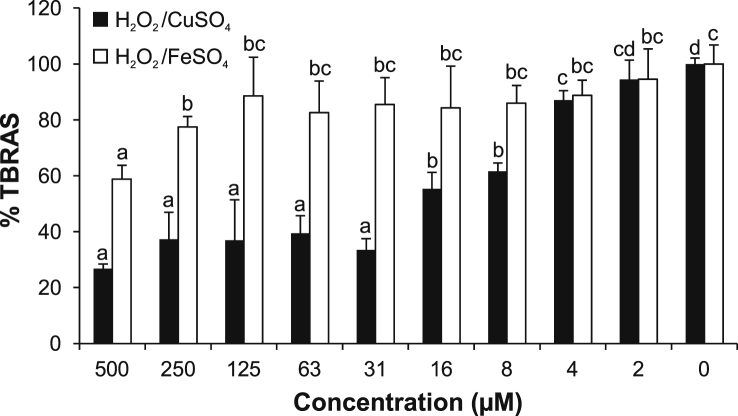
Effects of QUIN on the generation of thiobarbituric acid reactive species (TBARS) in the copper or iron catalyzed ascorbic acid-free variant of deoxyribose degradation assay. Error bars indicate the standard deviation of three replicates; letters (a–h) indicate different levels of significance (95% Duncan).

Both combinations of H_2_O_2_ with iron and copper generated TBARS from deoxyribose (Fig. 2). Increasing QUIN concentrations reduced the oxidative deoxyribose degradation differently if iron or copper were present in the reaction mixtures. If, however, QUIN was added as competitive ligand to the reaction solution, an antioxidant effect was more pronounced in case of copper than in case of iron. These results suggest that possible detrimental effects of copper can be quenched more efficiently than those of iron.

**Fig. 3:**
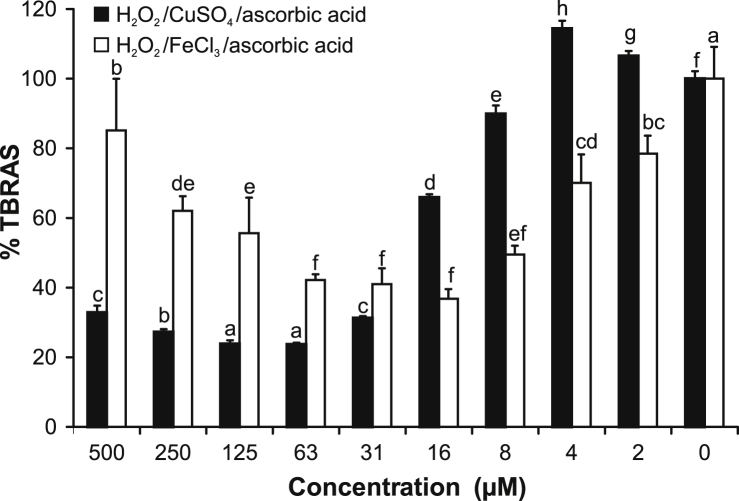
Effects of QUIN on the generation of thiobarbituric acid reactive species (TBARS) in the copper or iron catalyzed site-specific deoxyribose degradation assay. Error bars indicate the standard deviation of three replicates; letters (a–h) indicate different levels of significance (95% Duncan).

In the human brain, the common cellular antioxidant ascorbic acid can accumulate up to extracellular concentrations of 100–500 μM [18]. It can, however, start ROS production by enhancing of transition metal redox cycling [16]. Its presence reveals even more complex effects of QUIN (Fig. 3). Whereas increasing QUIN concentrations managed to quench the copper redox cycling more or less efficiently, this was not the case for iron. (The data for the iron catalyzed site-specific deoxyribose degradation assay were taken from Kubicova et al. 2013 [14].) The initial QUIN concentration increase managed only slightly to quench iron redox cycling. However, higher QUIN concentrations recovered iron redox cycling into a range comparing to the control without QUIN addition. If copper was present, only a slight pro-oxidant effect at lower concentrations appeared that, however, decreased with higher concentrations. The QUIN effect on the redox cycling of both metals was non-linear albeit more pronounced for iron than copper. In agreement with the assay exploring QUIN effects in the absence of ascorbic acid (Fig. 2), QUIN caused a stronger antioxidant effect if copper was the Fenton reaction catalyst. In contrast, Iwahashi et al. described the enhancing effect of QUIN on the Fenton reaction in phosphate buffer [19]. The different experimental setup used by these authors could provide a possible explanation for the observed enhancing effect of QUIN: Fe(NH_4_)_2_(SO_4_)_2_ was used as Fenton reaction catalyst and hydroxyl radical formation was detected by EPR. One of the known problems in studying the redox chemistry of biologically active substances is their highly milieu-dependent effects.

**Fig. 4:**
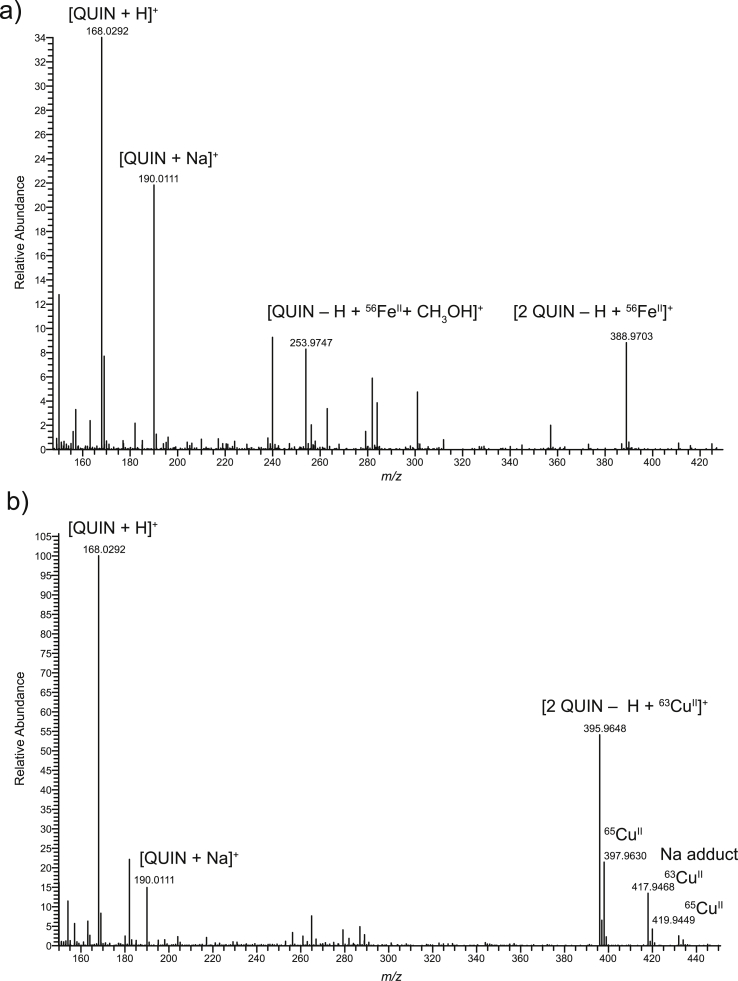
Nano-ESI–MS spectra in the positive mode of a) QUIN–iron(II) solution 3:1 and b) QUIN–copper(II) solution 2:1.

QUIN's ability of forming coordination complexes with iron(II) and copper(II) was explored by nano-ESI–MS technique in the positive ionization mode (Fig. 4). Nano-ESI–MS is a generally-used method for metal complex analyses because it informs directly about the stoichiometry of the coordination complexes in the solutions [17], [20], [21]. In addition, since the direct infusion method requires extremely small quantities of the analyzed samples, the danger of equipment contamination is dramatically decreased. In contrast to the separation methods, direct infusion technique can also avoid artifact formation arising by decomposition of instable substances [22].

The ESI-positive mass spectrum of iron(II) and QUIN mixtures (Fig. 4a) showed following ions: QUIN [M + H]^+^, *m/z* 168.0292, calc. theoretical mass 168.0291, C_7_H_6_NO_4_, and its Na-adduct of *m/z* 190.0111, calc. theoretical mass 190.0112, [M + Na]^+^, C_7_H_5_NNaO_4_. The ion detected at *m/z* 253.9747 (calc. molecular formula C_8_H_8_^56^Fe^II^NO_5_) was assigned to [M − H + ^56^Fe^II^ + CH_3_OH]^+^, theoretical mass 253.9747. The respective isotope pattern showed: C_8_H_8_^54^FeNO_5_, *m/z* 251.9794 (4% rel. int., calc. 6%, theoretical mass: 251.9794); C_8_H_8_^56^FeNO_5_ *m/z* 253.9747 (100% rel. int., calc. 100%); C_8_H_8_^57^FeNO_5_, *m/z* 254.9780 (6% rel. int., calc. 9%, theoretical mass: 254.9781); C_8_H_8_^58^FeNO_5_, *m/z* 255.9789 (1% rel. int., calc. 1%, theoretical mass: 255.9790).

A further ion was detected at *m/z* 388.9703 with a calc. molecular formula C_14_H_9_^56^FeN_2_O_8_. The following coordination complex ion could be assigned: [2M − H + ^56^Fe^II^]^+^, theoretical mass 388.9702. The respective isotope pattern showed: C_14_H_9_^54^FeN_2_O_8_, *m/z* 386.9742 (3% rel. int., calc. 6%, theoretical mass: 386.9749); C_14_H_9_^56^FeN_2_O_8_, *m/z* 388.9703 (100% rel. int., calc. 100%); C_14_H_9_^57^FeN_2_O_8_, *m/z* 389.9735 (10% rel. int., calc. 15%, theoretical mass: 389.9742); C_14_H_9_^58^FeN_2_O_8_, *m/z* 390.9734 (1% rel. int., calc. 3%, theoretical mass: 390.9749).

The mass spectrum of the copper(II) and QUIN mixture (Fig. 4b) showed the following ions: QUIN [M + H]^+^, *m/z* 168.0292; [M + Na]^+^, *m/z* 190.0111; *m/z* 395.9648 and *m/z* 397.9630 (calc. molecular formula C_14_H_9_^63^CuN_2_O_8_, theoretical mass 395.9648, rel. int. 100%, calc. 100%, and C_14_H_9_^65^CuN_2_O_8_, theoretical mass 397.9630, rel. int. 46%, calc. 45%), and *m/z* 417.9468 and 419.9449 (calc. molecular formulas C_14_H_8_^63^CuN_2_NaO_8_, theoretical mass 417.9468, rel. int. 100%, calc. 100%, and C_14_H_8_^65^CuN_2_NaO_8_, theoretical mass 419.9450, rel. int. 42%, calc. 46%), were attributed to the ions [2M − H + Cu^II^]^+^ and [2M − 2H + Cu^II^ + Na]^+^, respectively. On the contrary to the mass spectra of QUIN–Fe^II^ mixtures, no signals which could correspond to complexes with one QUIN molecule were identified in the mass spectrum of QUIN–Cu^II^ mixtures. All above mentioned peaks were detected in QUIN:metal(II) molar ratios 1:2, 1:1, 2:1, 3:1 and 4:1, but with different intensities (data not shown).

**Fig. 5:**
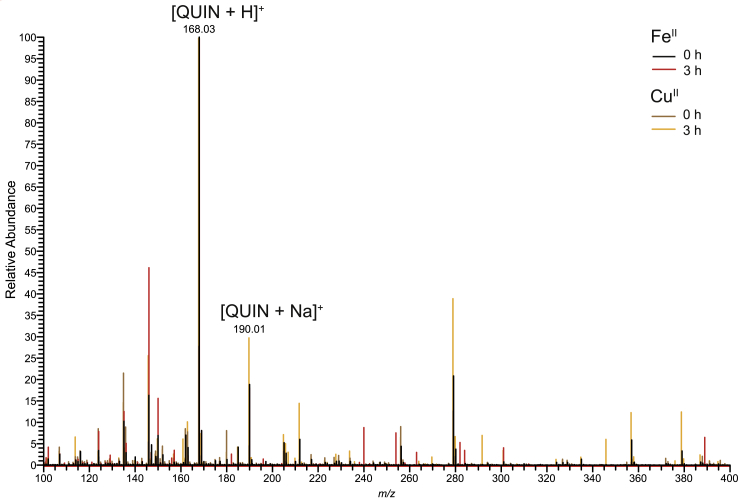
Nano-ESI–MS spectra in the positive mode of QUIN oxidation stability analysis in the presence of H_2_O_2_ and iron(II), t 0 h, black ions; H_2_O_2_ and copper(II), t 0 h, brown ions; H_2_O_2_ and iron(II), t 3 h, red ions; H_2_O_2_ and copper(II), t 3 h, orange ions.

The oxidation stability of QUIN was confirmed by nano-ESI–MS (Fig. 5). The mass spectra suggested that QUIN remained stable when attacked by hydroxyl radical. Fig. 5 demonstrates the similarity of the mass spectra of the reaction mixtures at the beginning of the incubation and after 3 h of incubation. These results provide evidence that QUIN does not reduce ROS levels by direct reaction with them. However, QUIN can show an antioxidant activity due to its effects on the redox cycling of the iron or copper central atoms in the complexes.

## Conclusions

Quinolinic acid is generally classified as an important endogenous neurotoxin which is involved in the development of various neurodegenerative processes. Its high concentrations that have been detected in brains of patients who suffer from neurodegenerative diseases [23] often correlate with elevated concentrations of the transition metals iron and copper [10]. In the plaques, QUIN probably forms coordination complexes with transition metals. Concomitantly, QUIN may decrease ROS production by inhibiting transition metal catalytic activity. Both properties can contribute to maintaining the important redox homeodynamic equilibrium. Changed reaction speeds that accompany aging possibly destroy this stabilizing capacity. The results of this study somehow question the general notion that QUIN is neurotoxic by revealing relatively complex milieu-dependent effects that depend also on QUIN's coordination chemistry.
